# Analysis of magnetic resonance splectroscopy characteristics in patients with type 2 diabetes complicated with stroke

**DOI:** 10.3389/fmed.2022.1008941

**Published:** 2022-11-04

**Authors:** Yu Wang, Ying Wang, Gang Peng, Wenwen Liang, Jie Chen, Kai Chen, Xiaodan Yang, Jiehui Jiang, Bingcang Huang

**Affiliations:** ^1^Department of Radiology, School of Medicine, Shanghai Pudong New Area Gongli Hospital, Shanghai University, Shanghai, China; ^2^Shanghai Health Commission Key Lab of Artificial Intelligence (AI)-Based Management of Inflammation and Chronic Diseases, Sino-French Cooperative Central Lab, School of Medicine, Shanghai Pudong New Area Gongli Hospital, Shanghai University, Shanghai, China

**Keywords:** ischemic stroke, hydrogen proton magnetic resonance imaging, glycosylated hemoglobin, NIHSS score, NAA/Cr

## Abstract

In this study, we investigated the metabolism of white matter by magnetic resonance spectroscopy (MRS) in stroke complicated with diabetes mellitus in combination with glycosylated hemoglobin (HbAlc) detection and clinical neurological deficit score (NIHSS). Fifty-three patients with stroke within 24 h after onset were collected and scanned by MRS. The biochemical, clinical and imaging characteristics of patients were analyzed. Patients were divided into three groups according to HbAlc levels: Good glycemic control (A): < 6.5%; satisfactory glycemic control (B): 6.5–7.5% and poor glycemic control (C): > 7.5%. The results showed that HbA1c levels were positively correlated with NIHSS in patients with acute ischemic stroke (AIS). There is significant difference in NAA/Cr between the infarcted site of the three groups and the mirror site. HbA1C level was negatively correlated with NAA/Cr in patients with AIS, and there was no significant correlation between NIHSS score and NAA/Cr. The data above demonstrated that the MRS imaging can be used to explain the adverse effects of hyperglycated hemoglobin on brain parenchyma from the perspective of imaging. This imaging technique and clinical NIHSS score have a high consistency in evaluating stroke.

## Introduction

Stroke is a cerebrovascular disease with high morbidity, mortality and disability. Diabetes is one of the independent risk factors for stroke (1–3). Diabetes patients are 2–6 times more likely to have a stroke than non-diabetics and the risk of recurrent stroke is also doubled (4, 5). Stroke patients combined with diabetes are more prone to recurrence and deterioration (4), and clinicians are required to urgently evaluate patients with appropriate imaging methods. Traditional neuroimaging techniques such as magnetic resonance imaging (MRI) and computed tomography (CT), can only show morphological changes after stroke (6) but cannot reveal the feature of nerves and biochemicals. In this study, magnetic resonance spectroscopy (MRS) was used to study the metabolism of white matter in patients with Type 2 diabetes Complicated with Stroke. Taking into consideration glycosylated hemoglobin and clinical neurological deficit score (NIHSS), biochemical and clinical analysis of diabetic patients who suffered a stroke was analyzed. By studying the characteristic evolution of the imaging, we can establish as early warning mechanism, which could eventually provide valuable information for the clinical treatment of cerebral infarction and for the evaluation of prognosis (7–9).

## Subjects and methods

### General information

We searched the database of Shanghai Pudong New Area Gongli hospital for patients who were admitted in the department of neurology between June 2015 to June 2017. The DWI imaging system had 53 patients with single stroke in the basal ganglia, which were included in the study. All patients included in this study were primary cerebral infarction. Inclusion criteria were as follows: Patients who met the 2014 edition of guidelines for the diagnosis and treatment of acute ischemic stroke (AIS) in China; exclusion criteria: hypertension, hyperlipidemia, brain injury patients and other neurological diseases, magnetic resonance imaging contraindications (patients with pacemakers, patients with nerve stimulators, artificial metal heart valves, intraocular metal foreign bodies, inner ear implants, *in vivo* ferromagnetic foreign bodies; early pregnancy—within first 3 months of pregnancy; severe Hyperthermia) and also patients who are claustrophobic.

Subjects signed informed consent.

### Instruments and methods

The TOSHIBA EXCELART Vantage 1.5T magnetic resonance imaging instrument is equipped with an 8-channel head and neck coil. All subjects underwent routine MRI (T1WI, T2WI, FLAIR) sequence, diffusion weighed imaging (DWI) and MRS examination.

DWI was as follows: SE sequence, TE1550 ms, matrix 128 × 128, FOV 24 × 24, layer thickness 5.5 mm, layer spacing 1.0 mm, *b*-value 1,000 s/mm^2^.

MRS characteristics was as follows: Multi-voxel acquisition using point-resolved spectroscopy (PRESS). General parameters: TR 1,500 ms, TE 136 ms, average number of 1, and detection time: 6:15 min.

Volume of interest (VOI) Selection: According to the high sensation area of the basal ganglia on the DWI, the acute infarct area was used as the VOI. The sampling volume is based on 15 mm × 15 mm × 15 mm. According to the size of the lesion to be detected, the lesion area should be included in one voxel as much as possible, and we tried to avoid the interference of the skull, fatty tissue and cerebrospinal fluid.

Within 24 h of admission, the author used the National Institutes of Health Stroke Scale (NIHSS score) to score neurological deficits in patients. The grading was done as follows: mild impairment: NIHSS ≤ 6 points, moderate impairment: 6–16 points, severely affected: ≥ 16 points. As far as possible, we did not repeat our instructions or make the patients do the same movement twice (such as repeatedly asking the patient to do some kind of effort). The time taken to score the patient was not more than 2 min.

Venous blood was collected within 24 h after admission, and glycated hemoglobin (HbA1c) was detected by high pressure liquid chromatography. According to the 2013 American Diabetes Association, Diagnosis and classification of diabetes mellitus (7), HbA1C ≥ 6.5% is listed as Diagnostic criteria for diabetes. Blood glucose control is grouped according to glycated hemoglobin levels. Good glycemic control group (group A): HbAlc < 6.5%; Satisfactory glycemic control group (group B): HbAlc: 6.5∼7.5%; poor glycemic control group (group C): HbAlc > 7.5%.

### Statistical analysis

The data was analyzed using statistical software SPSS22.0. The *t*-test and analysis of variance were used to compare the general data, NIHSS score and MRS parameters of the three groups. The correlation between NIHSS score and HbAlc, the correlation between NIHSS score and MRS parameters of the three groups were analyzed by Pearson correlation (when | r| ≥ At 0.8, it was considered that the two variables are highly correlated; when 0.5 ≤ | r| ≤ 0.8, the two variables were considered to be moderately related; when 0.3 ≤ | r| ≤ 0.5, the two variables were considered to have a low correlation. When 0 ≤ | r| ≤ 0.3, the degree of correlation is weak and basically irrelevant). *P* < 0.05 was considered statistically significant.

## Results

### General information comparison

The clinical characteristics of 53 stroke patients in this study are shown in Table 1. There were no significant differences in gender and mean age between the three groups (*P* = 0.1, *P* = 0.5). The three groups were investigated for metabolic indicators such as low-density lipoprotein, fasting blood glucose, and HbAlc. The differences were statistically significant (*P* < 0.05, *P* < 0.01, *P* < 0.01).

**TABLE 1:** Clinical characteristics of 53 stroke patients.

Indicator (Mean/Range)	Stroke patients (*n* = 53)		
	
	Good glycemic control group (Group A)(*n* = 31)	Satisfactory glycemic control group (Group B)(*n* = 9)	Poor glycemic control group (Group C)(*n* = 13)
Sex (Male/Female)	21/10	5/4	7/6
Age (years)^a^	70.2/49–90	71.6/60–84	67.5/46–87
LDL-C (mmol/L)^a^	2.63/1.05–4.61	2.91/1.17–4.89	3.42/1.67–5.44
FBG (mmol/L)^a^	5.16/3.77–6.91	5.90/4.63–7.60	9.29/4.99–16.89
HBA1C (%)^a^	5.8/5.0–6.3	6.8/6.5–7.2	9.7/7.6–13.2

LDL-C, low density cholesterol; FBG, Fasting blood sugar; HBA1C, glycosylated hemoglobin. ^a^Median (range).

### Neurological function score

The NIHSS scores of the three groups are shown in Table 2. The NIHSS scores of the three groups were compared. Group A: 3.6129 ± 3.56537, Group B: 4.6667 ± 2.59808, Group C: 6.4615 ± 2.81707, the difference was statistically significant (*P* < 0.05).

**TABLE 2:** NIHSS scores of three groups of patients.

	≤6 points	6∼16 points	≥16 points	Range	Median
Group A (*n* = 31)	26	3	1	0∼16	2
Group B (*n* = 9)	7	2	0	0∼9	5
Group C (*n* = 13)	7	6	0	0∼12	6

NIHSS, National Institutes of Health Stroke Scale; Group A, Good glycemic control group, HbAlc < 6.5%; Group B:Satisfactory glycemic control group, HbAlc: 6.5% 7.5%; Group C: Poor glycemic control group, HbAlc > 7.5%. Range: the highest and lowest of the NIHSS scores of each group of patients; Median: the median NIHSS score of each group.

**FIGURE 1:**
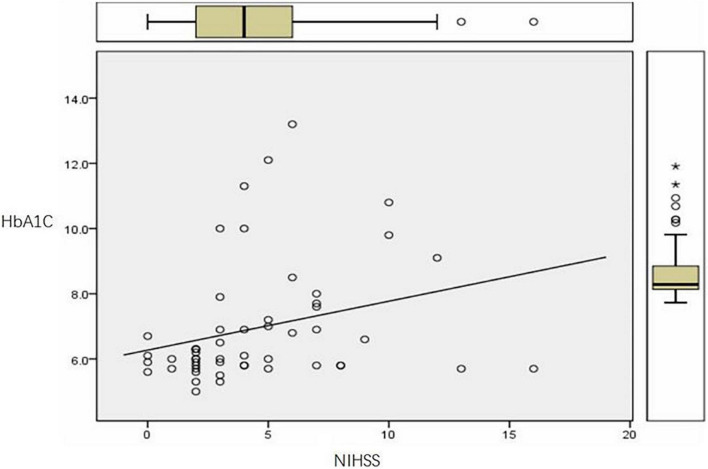
HbA1C and NIHSS diagram.

HbA1C levels and NIHSS scores of the three groups A, B, C were compared, Pearson correlation analysis showed that patients with AIS had a positive correlation between blood HbA1C levels and NIHSS (*r* = 0.276, *P* ≤ 0.05), that is, the higher the HbA1C level, the higher the NIHSS score (Figure 1).

### Changes in magnetic resonance spectroscopy metabolites

In group A, B, C, the metabolites of the site of infarcts and their contralateral mirror site were compared (see Table 3). In Group A infarct side NAA/Cr: 1.09 ± 0.22, mirror side NAA/Cr: 1.67 ± 0.36, *t* = –7.647; Group B infarct side NAA/Cr: 0.91 ± 0.14, mirror side NAA/Cr: 1.16 ± 0.24, *t* = –2.618; Group C infarct side NAA/Cr: 0.87 ± 0.21, mirror side NAA/Cr: 1.16 ± 0.26, *t* = –3.098, the difference was statistically significant (*P* < 0.01, < 0.05, < 0.05), and for the Cho/Cr ratio the difference was not statistically significant (*t* = –2.261, –1.842, –4.522, *P* < 0.05, = 0.05, < 0.05), Lac appeared 25 times in total.

**TABLE 3:** Characteristics of metabolites following brain infarct for the three groups.

Metabolites χ ± S	Group A (*n* = 31)			Group B (*n* = 9)			Group C (*n* = 13)		
			
	IS	MI	*P*-value	IS	MI	*P*-value	IS	MI	*P*-value
NAA/Cr	1.09 ± 0.22	1.67 ± 0.36	0.007**	0.91 ± 0.14	1.16 ± 0.24	0.038*	0.87 ± 0.21	1.16 ± 0.26	0.029*
Cho/Cr	0.87 ± 0.19	1.04 ± 0.38	0.039*	0.79 ± 0.21	1.07 ± 0.41	0.036*	0.74 ± 0.19	1.3 ± 0.40	0.030*
Lac/Cr	0.35 ± 0.10	0	/	0.38 ± 0.13	0	/	0.49 ± 0.12	0	/

Group A, Good glycemic control group, HbAlc < 6.5%; Group B: Satisfactory glycemic control group, HbAlc: 6.5% 7.5%; Group C, Poor glycemic control group, HbAlc > 7.5%; IS: Infarcted area side; MI, Mirror of infarcted area; NAA, N-acetyl aspartate; Cr, Creatine; Cho, Choline; Lac, Lactate.

*P < 0.05, **P < 0.01.

Comparison of metabolites between infarcts in patients of group A, B and C: There was a significant difference in NAA/Cr (*P* < 0.01) but there was no significant difference in Cho/Cr (*P* > 0.05). The difference in Lac/Cr was statistically significant (*P* < 0.05).

**FIGURE 2:**
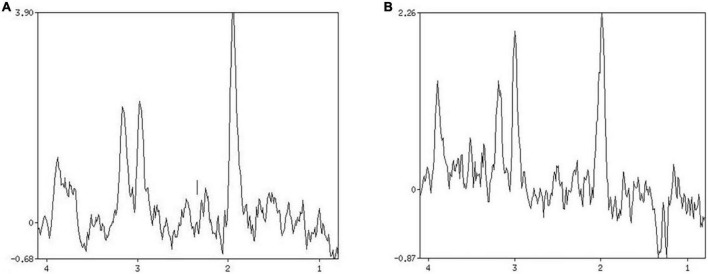
Patient 1 HbAlc: 5.7. **(A)** MRS shows changes in brain metabolites in the infarcted area, NAA/Cr 2.334, Cho/Cr 1.332. **(B)** MRS shows changes in brain metabolites in the contralateral side at the same site, NAA/Cr 1.780, Cho/Cr 0.985.

**FIGURE 3:**
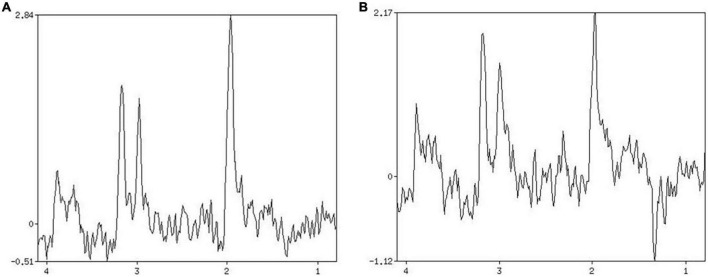
Patient 2 HbAlc: 10.3. **(A)** MRS shows changes in brain metabolites in the infarcted area, NAA/Cr 1.906, Cho/Cr 0.926. **(B)** MRS shows changes in brain metabolites in the contralateral side at the same site, NAA/Cr 1.422, Cho/Cr 1.571.

The blood HbA1C and NIHSS scores of patients in group A, B and C were compared with respect to NAA/Cr, Cho/Cr and Lac/Cr values. Pearson correlation analysis showed that blood HbA1C levels had a low negative correlation with NAA/Cr (*r* = –0.494, *P* ≤ 0.01, it had a low negative correlation with Cho/Cr (*r* = –0.354, *P* ≤ 0.01), and the correlation with Lac/Cr was not statistically significant (*r* = 0.252, *P* = 0.1). There was no significant correlation between NIHSS score and NAA/Cr, Cho/Cr, and Lac/Cr (*r* = –0.135, *r* = –0.004, *r* = 0.164, *P* = 0.1) (Figures 2, 3).

## Discussion

### Application of magnetic resonance spectroscopy in stroke patients

The 1H-MRS detection index usually includes nitrogen-acetylaspartate (NAA), choline complex (Cho), creatine (Cr), and lactic acid (Lac) (10, 11). The ratio of NAA/Cr and Cho/Cr reflects the change of NAA and Cho concentration to some extent. To a certain extent, it can be used for the diagnosis and evaluation of diseases (12–14).

In this experiment, 53 patients with acute cerebral infarction were selected and the proton magnetic resonance spectroscopy (1H-MRS) imaging method was used to image the cerebral infarction lesions, the metabolites in the lesions and their clinical value. In the 53 patients who underwent MRS imaging, the NAA concentration in the infarct site was found to be decreased, and the results were consistent with the literature reports. The concentration of Cho was not significantly changed. The typical Lac peak was only found 23 times, and the incidence rate was 43.4%. This may be due to the fact that the concentration of Lac is related to anaerobic metabolism. When the examination time exceeds the acute phase of stroke, the blood vessels around the hypoxic brain tissue revascularize and the oxygen supply increases, so Lac cannot be detected. In addition to this, when the total amount of Lac is small, contamination due to Lipid peak also reduces the detection rate of Lac.

### Characteristics of magnetic resonance spectroscopy images of stroke patients complicated with type 2 diabetes

Cerebrovascular disease is a chronic complication of diabetes and one of the leading causes of death in diabetic patients; hyperglycemia is also an independent risk factor for stroke (15–18).

The infarction site of diabetic patients with stroke is common in basal ganglia, corona radiata and brain lobe. The infarct type is mainly small infarct, and can also be found in the thalamus, quadrigemina, lateral ventricle and other parts. In order to reduce the error caused by the different content of metabolites in different measurement sites, the basal ganglia infarct was selected as the measurement site. Comparing the MRS images of the basal ganglia infarction area of the three groups of patients, we found that the NAA/Cr value of the satisfactory glycemic control group was not significantly different from that of the good glycemic control group, while the NAA/Cr value of the poor glycemic control group was significantly lower than that of the good glycemic control group. The Cho/Cr index did not show a significant difference between the three groups. There was no significant difference in the appearance of Lac peak between the three groups. In the observed Lac peak, Lac/Cr was significantly higher in the poor glycemic control group as compared to the good glycemic control group, but the correlation was not obvious. This may be related to the small number of observations of the Lac peak and the lower concentration of Lac. At the same time, we also found that even in the area where no infarction occurred, the NAA/Cr value of the poor glycemic control group was lower than that of the good glycemic control group, which further indicates that the blood sugar elevation aggravates the nerve fiber damage and anaerobic metabolism. Therefore, Stroke patients who are also diabetic often have more severe clinical symptoms and a poorer prognosis.

NAA is a marker of neuronal cell integrity. Our study found that blood HbA1C levels were negatively correlated with NAA/Cr in patients with AIS, i.e., the higher the glycated hemoglobin level, the greater the destruction of neuronal cell integrity. Cho reflects the renewal state of the cell membrane. This experiment found that the level of HbA1C was negatively correlated with Cho/Cr, that is, high levels of HbA1C slowed down cell renewal and decreased cell viability. These results further confirm the fact that an increase in blood glucose causes a decrease in neuronal activity and also causes nerve fiber damage.

The increase in HbA1c leads to an increase in the incidence of stroke, the disease has an increased severity and this may be due to: (1) effects of long-term hyperglycemia, hyperglycemia end products accumulate in the patient, leading to thickening, damage, and stimulation of the inner wall of the blood vessel. The release of inflammatory cytokines promotes the formation of atherosclerosis and cerebral arterial thrombosis, which ultimately induces stroke; (2) hyperglycemia reduces the formation of new blood vessels through the action of low vascular endothelial growth factor, inducing a reduced perfusion in the periphery of the lesions and lactic acid accumulation in the brain also promotes the development of hypoperfusion which causes irreversible infarction and which therefore accelerates the pathological process of the lesion; (3) microvascular dysfunction caused by diabetes, vascular endothelial proliferation, increased blood viscosity, decreased blood flow velocity, red blood cells and platelet aggregation occur which then leads to the formation of thrombus which further increases the incidence and recurrence rate of stroke.

### Correlation between magnetic resonance spectroscopy parameters and national institutes of health stroke scale scores

The National Institutes of Health Stroke Scale (NIHSS) is a clinically used scale for assessing neurological deficits in patients with acute stroke. It has become an important part of the clinical evaluation and clinical trial system for acute stroke. The standard NIHSS consists of 15 items, including language, motor function, sensory function, and movement. The score ranges from 0 to 42. A score < 6 points indicate a good prognosis. A score > 16 points indicate the possibility of death and severe disability.

No significant correlation was observed between NIHSS score and NAA/Cr, Cho/Cr, and Lac/Cr detected by MRS in this study. The NIHSS score is a result of clinicians’ evaluation of patients, as such it is subjective and cannot completely reflect the neurological impairment of patients. MRS is more sensitive and can detect microscopic changes in substances, but these small change does not necessarily affect the patient’s clinical symptoms, that is, “only qualitative changes occur, without quantitative changes occur.” We therefore need to question whether these is a threshold for symptomatic change to occur. When the concentration of substances detected by MRS exceeds a certain threshold, this could potentially a greater impact on the NIHSS score, which needs to be studied further. The equations should be inserted in editable format from the equation editor.

## Conclusion

Magnetic resonance spectroscopy is the only non-invasive imaging method for studying the metabolism, biochemical changes and for quantitative analysis of compounds in living organs. NAA/Cr, Cho/Cr, and Lac/Cr are quantitative indicators that show the changes in brain chemical metabolism in stroke patients after the onset, providing a direct imaging basis for the severity and prognosis in stroke patients. Elevated blood glucose is an important risk factor for stroke and a major cause of increased mortality among stroke patients. Using MRS imaging method, the damage to the brain caused by hyperglycated hemoglobin is explained from the perspective of microscopic substance changes. This technique helps to better assist in the early clinical diagnosis and prognostic assessment of stroke disease.

The shortcomings of this study are as follows: 1. MRS examination time is too long, the movement of patients with acute stroke will increase the chance of artifacts, the signal is weak, the spatial resolution is poor and its application in acute stroke still requires a better understanding and further improvement; 2. Due to the fact that the sample size is not big enough, the research on the changes in Cho value in infarcted lesion area is therefore not complete. Follow-up studies on this matter will need to expand the sample size and improve the detection of Cho and add additional observation indicators to the study.

## Data availability statement

The original contributions presented in this study are included in the article/supplementary material, further inquiries can be directed to the corresponding author.

## Ethics statement

The studies involving human participants were reviewed and approved by the Ethics Committee of Gongli Hospital of Shanghai Pudong New Area. The patients/participants provided their written informed consent to participate in this study.

## Author contributions

YuW, YiW, and GP: conceptualization and writing—original draft preparation. WL and JC: data curation. KC and XY: investigation. JJ: supervision. BH: supervision and writing—review and editing. All authors approved the submitted manuscript.
